# A Lassa Fever Live-Attenuated Vaccine Based on Codon Deoptimization of the Viral Glycoprotein Gene

**DOI:** 10.1128/mBio.00039-20

**Published:** 2020-02-25

**Authors:** Yingyun Cai, Chengjin Ye, Benson Cheng, Aitor Nogales, Masaharu Iwasaki, Shuiqing Yu, Kurt Cooper, David X. Liu, Randy Hart, Ricky Adams, Tyler Brady, Elena N. Postnikova, Jonathan Kurtz, Marisa St Claire, Jens H. Kuhn, Juan Carlos de la Torre, Luis Martínez-Sobrido

**Affiliations:** a Integrated Research Facility at Fort Detrick, National Institute of Allergy and Infectious Diseases, National Institutes of Health, Fort Detrick, Frederick, Maryland, USA; b Department of Microbiology and Immunology, University of Rochester, Rochester, New York, USA; c Department of Immunology and Microbiology, The Scripps Research Institute, La Jolla, California, USA; Icahn School of Medicine at Mount Sinai

**Keywords:** *Arenaviridae*, arenavirid, arenavirus, *Bunyavirales*, bunyavirus, guinea pig, Lassa, Lassa fever, Lassa virus, LASV, LAV, live-attenuated vaccine, mammarenavirus, strain 13, vaccine, VHF, viral hemorrhagic fever

## Abstract

Lassa virus (LASV) is endemic in Western Africa and is estimated to infect hundreds of thousands of individuals annually. A considerable number of these infections result in Lassa fever (LF), which is associated with significant morbidity and a case-fatality rate as high as 69% among hospitalized confirmed patients. U.S. Food and Drug Administration-approved LF vaccines are not available. Current antiviral treatment is limited to off-label use of a nucleoside analogue, ribavirin, that is only partially effective and associated with significant side effects. We generated and characterized a recombinant LASV expressing a codon-deoptimized (CD) glycoprotein precursor gene (GPC), rLASV-GPC/CD. Comparison of growth kinetics and peak titers showed that rLASV-GPC/CD is slightly attenuated in cell culture compared to wild-type (WT) recombinant LASV (rLASV-WT). However, rLASV-GPC/CD is highly attenuated in strain 13 and Hartley guinea pigs, as reflected by the absence of detectable clinical signs in animals inoculated with rLASV-GPC/CD. Importantly, a single subcutaneous dose of rLASV-GPC/CD provides complete protection against an otherwise lethal exposure to LASV. Our results demonstrate the feasibility of implementing a CD approach for developing a safe and effective LASV live-attenuated vaccine candidate. Moreover, rLASV-GPC/CD might provide investigators with a tool to safely study LASV outside maximum (biosafety level 4) containment, which could accelerate the elucidation of basic aspects of the molecular and cell biology of LASV and the development of novel LASV medical countermeasures.

## Abstract (precis)

Lassa virus (LASV) infects several hundred thousand people in Western Africa, resulting in many lethal Lassa fever (LF) cases. Licensed LF vaccines are not available, and anti-LF therapy is limited to off-label use of the nucleoside analog ribavirin with uncertain efficacy. We describe the generation of a novel live-attenuated LASV vaccine candidate. This vaccine candidate is based on mutating wild-type (WT) LASV in a key region of the viral genome, the glycoprotein precursor (GPC) gene. These mutations do not change the encoded GPC but interfere with its production in host cells. This mutated LASV (rLASV-GPC/CD) behaves like WT LASV (rLASV-WT) in cell culture, but in contrast to rLASV-WT, does not cause disease in inoculated guinea pigs. Guinea pigs immunized with rLASV-GPC/CD were protected against an otherwise lethal exposure to WT LASV. Our results support the testing of this candidate vaccine in nonhuman primate models ofLF.

## INTRODUCTION

Lassa virus (LASV), the causative agent of Lassa fever (LF), is endemic in Western Africa (1). LASV is estimated to infect hundreds of thousands of individuals annually, with about 20% of infected individuals developing clinical symptoms and signs, and is associated with a high case-fatality rate (CFR) (2). The majority of human infections occur after direct contact with infected rodents (predominantly *Mastomys* spp.) or their excreta (3). Areas where LASV is endemic cover large regions within Western Africa, with an at-risk population as high as 200 million people (4). Evidence indicates that regions where LASV is endemic are expanding (5), and the high degree of LASV genetic diversity likely contributes to underestimating its prevalence (6). Moreover, imported cases of LF have been reported in the United States, Canada, and Europe, including two recent exported cases of LF from Sierra Leone to the Netherlands in November 2019 (7–10), suggesting that local outbreaks could expand globally. To date, U.S. Food and Drug Administration-approved LASV vaccines are not available, and current anti-LASV therapy is limited to the use of ribavirin, which is only partially effective and can cause significant side effects (11, 12). The impact of LF on human health and the limited existing countermeasures to combat LF resulted in the inclusion of LF on the revised list of priority diseases for the World Health Organization (WHO) R&D Blueprint (13–16).

LASV is an Old World mammarenavirus (*Bunyavirales*: *Arenaviridae*) (17). Mammarenaviruses have bisegmented single-stranded RNA genomes and produce enveloped virions (18). Each genome segment contains two open reading frames in ambisense direction separated by a noncoding intergenic region (18). The large (L) genome segment encodes the large protein (L) that has RNA-directed RNA polymerase activity, and the RING finger protein Z, which functions as a matrix-like protein for virion assembly and budding (19–22). The small (S) genome segment encodes the nucleoprotein (NP) and the glycoprotein precursor (GPC) (23, 24). NP is the most abundant viral protein both in infected cells and in virions and is the main structural component of viral ribonucleoprotein (RNP) complexes. RNPs direct viral RNA (vRNA) genome replication and gene transcription (25). NP also plays an important role in counteracting host-cell innate immune responses (26–29). GPC is cotranslationally processed by cellular signal peptidase to generate a stable signal peptide (SSP) and posttranslationally cleaved by the cellular proprotein convertase subtilisin kexin isozyme-1/site-1 protease (SKI-1/S1P) to generate GP1 and GP2 subunits (30). GP1 and GP2, together with SSP, form the mature glycoprotein (GP) peplomers on the surface of the virion envelope that mediate virion cell entry via receptor-mediated endocytosis (31, 32).

The mammalian genetic code is degenerated, with most amino acids being coded by multiple synonymous codons. Although synonymous codons have the same coding potential, most organisms exhibit differences in the frequency at which they use synonymous codons to incorporate the same amino acid residue into a protein (33–36), a phenomenon called codon usage bias (37, 38). This bias is the basis for codon optimization or codon deoptimization (CD) experimental strategies to increase or decrease, respectively, gene expression in different organisms. CD is achieved by replacing wild-type (WT) codons for those with less-preferred codons throughout a target gene without affecting the amino acid sequence of the corresponding protein (35, 39). To date, several attenuated viruses, including poliovirus (40), human respiratory syncytial virus (41), foot-and-mouth disease virus (42), influenza A virus (43), and Zika virus (44) have been generated using a CD strategy.

We document that a recombinant form of the mammarenavirus lymphocytic choriomeningitis virus (rLCMV) with a CD GPC (rLCMV-GPC/CD) has growth properties similar to those of its WT counterpart (rLCMV-WT) in cultured cells. However, rLCMV-GPC/CD is highly attenuated in a laboratory mouse model of LCMV infection (45). Importantly, immunization of laboratory mice with a single dose of rLCMV-GPC/CD provided complete protection against an otherwise lethal infection with rLCMV-WT (45). These results led us to examine whether the same findings could be extended to LASV. In the present work, we document the generation and characterization of a rLASV expressing a CD GPC, rLASV-GPC/CD. Compared to rLASV-WT, rLASV-GPC/CD was significantly impaired in GP expression in infected cells, which surprisingly resulted only in a slight decrease in viral fitness compared to rLASV-WT. Importantly, rLASV-GPC/CD was completely attenuated in strain 13 and Hartley guinea pigs and provided full protection, upon a single administration, against an otherwise lethal exposure to LASV.

## RESULTS

### Codon deoptimization results in reduced LASV GPC expression.

**FIG 1:**
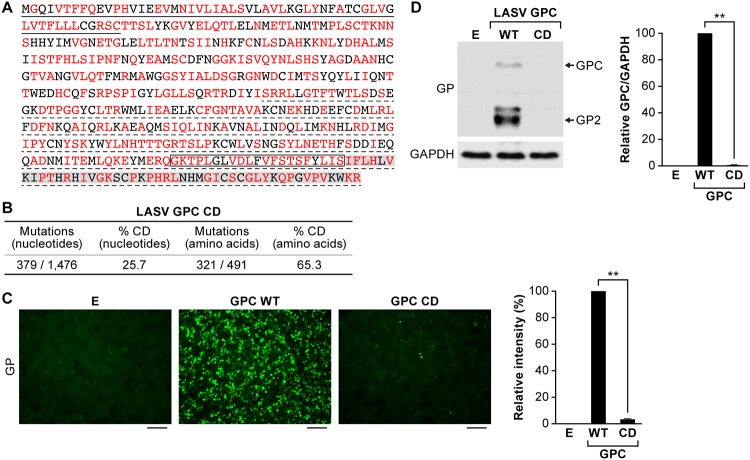
Codon deoptimization results in reduced LASV GPC expression. (A) Amino acid sequence of LASV GPC. Amino acid residues encoded by altered synonymous codons in the LASV GPC gene are indicated in red. Underlined (solid) amino acid residues indicate the LASV GPC SSP. The region that is not underlined corresponds to the GP1 subunit of LASV GPC, whereas underlined (dotted) amino acid residues correspond to the GP2 subunit of LASV GPC. A box indicates the LASV GP2 transmembrane domain. Amino acid residues highlighted in gray correspond to the GP2 cytoplasmic tail. (B) Numbers of nucleotide and amino acid residues affected by CD. (C and D) LASV GP expression in HEK293T/17 cells transfected with empty (lane E), LASV GPC WT or LASV GPC CD plasmids at 48 h p.t., as evaluated by IFA (C) or WB (D). The cross-reactive anti-LCMV GP2 MAb 83.6 detects both LASV GPC and GP2. The additional bands in the WB correspond to different glycosylated forms of GP2. Detection of GAPDH with an anti-GAPDH antibody served as a loading control in the WB assay. The intensity of fluorescence in the images shown in panel C was quantitatively analyzed by ImageJ. The relative intensity was calculated by normalizing to fluorescence intensity of LASV GPC WT. The bands of LASV GPC and GP2 shown in panel D were quantitatively analyzed by ImageJ, and the relative intensity of LASV GPC/GAPDH was calculated by normalizing to the ratio of WT GPC/GAPDH. IFA images and WB results are representative of three independent transfection experiments. Scale bars, 100 μm. **, *P* < 0.01 (Student *t* test).

We generated CD LASV GPC by *de novo* synthesis of a WT LASV GPC gene in which 379 synonymous nucleotide mutations were introduced to incorporate the least frequently used codon in mammalian cells (39) for 321 of 491 (65%) amino acid residues (Fig. 1A and B; see also Fig. S1 and Supplemental Methods S1 in the supplemental material). Expression of LASV GP was 50- to 100-fold lower in HEK293T/17 cells transfected with pCAGGS encoding LASV GPC CD than in the LASV GPC WT, as determined by immunofluorescence assay (IFA) (Fig. 1C) or Western blotting (WB) (Fig. 1D).

10.1128/mBio.00039-20.2 FIG S1 Nucleotide sequences of LASV GPC WT and GPC/CD. LASV GPC WT sequence is highlighted in gray (top rows). LASV GPC/CD is indicated in the second rows. Nucleotide substitutions for CD are indicated in red. Download FIG S1, TIF file, 1.0 MB. Copyright © 2020 Cai et al. 2020 Cai et al. This content is distributed under the terms of the Creative Commons Attribution 4.0 International license.

### Generation of rLASV-GPC/CD.

We used an LASV reverse genetics system in which the T7 RNA polymerase promoter directs the synthesis of L and S segment vRNAs (Fig. S2A). HEK293T/17 cells were transfected with the indicated set of five plasmids (Fig. S2A and Supplemental Methods S1). At day 3 posttransfection (p.t.), tissue culture supernatants (TCS) were collected, and fresh media were added to the transfected cells. On day 6 p.t., TCS (P0D6) were collected, and transfected HEK293T/17 cells were cocultured with fresh Vero cells. After day 10 p.t. (day 4 of coculture), TCS (P0D10) were collected, and fresh media were added. After day 13 p.t. (day 7 of coculture), TCS (P0D13) were collected, and viral titers in the TCS were determined for all three time points by plaque assay (Fig. S2B). At day 6 p.t., only a low titer of rLASV-GPC/CD (7.33 × 10^1^ PFU/ml) was detected in TCS. However, coculture of transfected HEK293T/17 cells with Vero cells amplified the titer of rLASV-GPC/CD dramatically (7.83 × 10^5^ PFU/ml).

10.1128/mBio.00039-20.3 FIG S2 Generation of rLASV-GPC/CD. (A) Rescue strategy. pT7-LASV-Sag and pT7-LASV-Lag direct synthesis of the LASV S and L segment RNAs (antigenome polarity), respectively, each under a T7 RNA polymerase promoter. Support plasmids pCAGGS-LASV-L and pCAGGS-LASV-NP express LASV L and NP, respectively. pCAGGS-T7 pol expresses T7 RNA polymerase. pT7-LASV-Sag/GPC/CD encoding the LASV GPC CD sequence was used instead of pT7-LASV-Sag to rescue rLASV-GPC/CD. HEK293T/17 cells were cotransfected with five plasmids as indicated. TCS were collected, and transfected cells were detached and cocultured with fresh Vero cells. TCS were harvested on day 10 (P0D10) and day 13 (P0D13) p.t. HDV, hepatitis delta virus ribozyme (HDV-Rbz) sequence; pA, poly(A) tail. (B) Virus titers of rLASV-WT and rLASV-GPC/CD in TCS at the indicated days were measured by plaque assay. Download FIG S2, TIF file, 1.1 MB. Copyright © 2020 Cai et al. 2020 Cai et al. This content is distributed under the terms of the Creative Commons Attribution 4.0 International license.

### Characterization of rLASV-GPC/CD.

**FIG 2:**
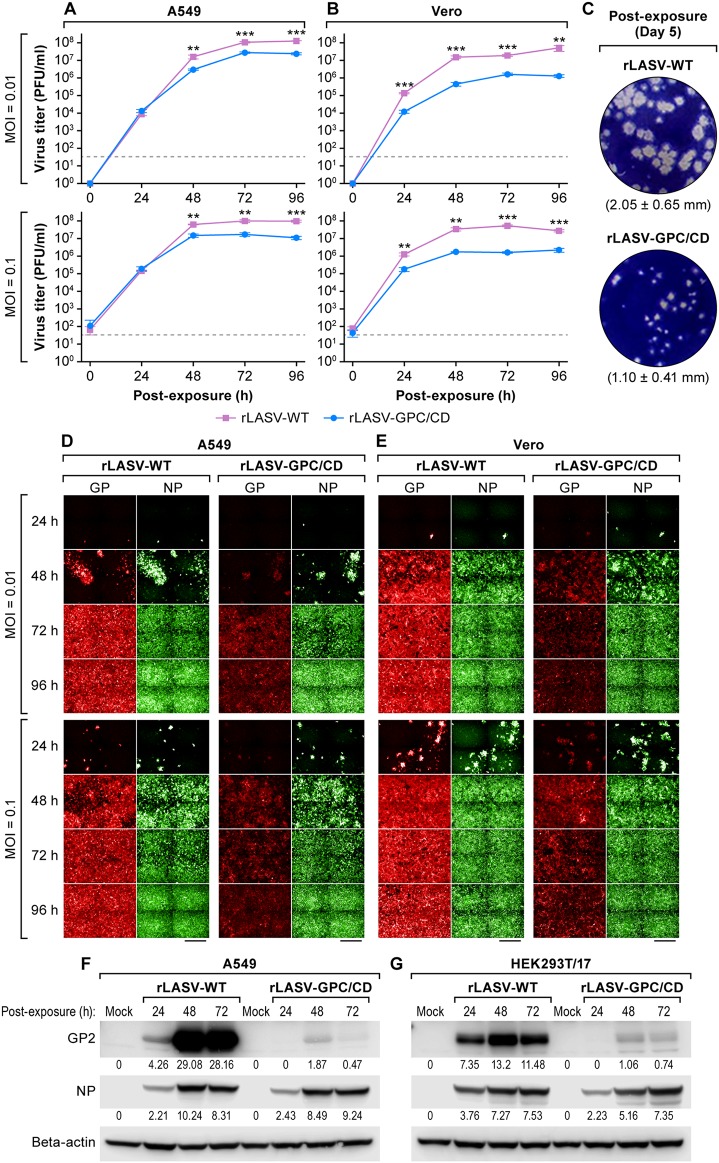
Characterization of rLASV-GPC/CD. (A and B) A549 and Vero cells were inoculated with rLASV-WT or rLASV-GPC/CD at the indicated MOIs, and viral titers in TCS were determined by plaque assay at the indicated time points. Dotted lines indicate the limit of detection (33 PFU/ml). Data represent the means ± the standard deviations (SD) of triplicate samples. **, *P < *0.01; ***, *P < *0.001 (Student *t* test). (C) Plaque morphologies and size of rLASV-WT and rLASV-GPC/CD on Vero cell monolayers. Plaque sizes presented are means ± SD of 25 randomly selected plaques. (D and E) Replicates of cells infected as before were evaluated for LASV NP and GP expression by IFA using anti-LASV NP and GPC MAbs. After IFA staining, images were collected at ×20 magnification from nine fields in each well. Representative images of four adjacent fields from one well are illustrated. Scale bars, 200 μm. (F and G) WB analysis of LASV NP and GP expression in A549 and Vero cells inoculated with rLASV-WT or rLASV-GPC/CD (MOI = 0.1). Numbers below the bands correspond to densitometry quantification (i.e., the optical density) of each band normalized to actin beta.

The growth kinetics of rLASV-WT and rLASV-GPC/CD in interferon (IFN)-competent (A549) and IFN-deficient (Vero) cells were compared using different multiplicities of infection (MOIs) (Fig. 2). rLASV-GPC/CD replicated efficiently in both cell types, although viral peak titers in both cell lines were up to an order of magnitude lower than those of rLASV-WT (Fig. 2A and B). Plaques induced by rLASV-GPC/CD (1.10 ± 0.41 mm) were smaller than those caused by rLASV-WT (2.05 ± 0.65 mm) (*n* = 25, *P* < 0.001; Fig. 2C). The temporal expression of LASV GP and NP was analyzed by IFA (Fig. 2D and E). NP expression was similar in both rLASV-WT- and rLASV-GPC/CD-infected cells, whereas GP expression was significantly lower in rLASV-GPC/CD-infected cells compared to rLASV-WT. Similarly, reduced GP expression was noted by WB in both cell types infected with rLASV-GPC/CD (Fig. 2F and G). Taken together, these results indicate that replication of rLASV-GPC/CD is slightly reduced compared to that of rLASV-WT, likely due to reduced GP expression.

### rLASV-GPC/CD is attenuated in strain 13 guinea pigs.

Strain 13 (inbred) and Hartley (outbred) guinea pigs have been used extensively to study LASV pathogenesis and to evaluate the efficacy of candidate therapeutics and vaccines (46, 47). Strain 13 guinea pigs are highly sensitive to LASV infection, with 100% lethality following subcutaneous (s.c.) inoculation of 10^3^ PFU, whereas Hartley guinea pigs are more resistant (30% lethality following s.c. inoculation of 2.4 × 10^5^ PFU) (48). Considering this difference, the safety profile of rLASV-GPC/CD was evaluated in strain 13 guinea pigs.

**FIG 3:**
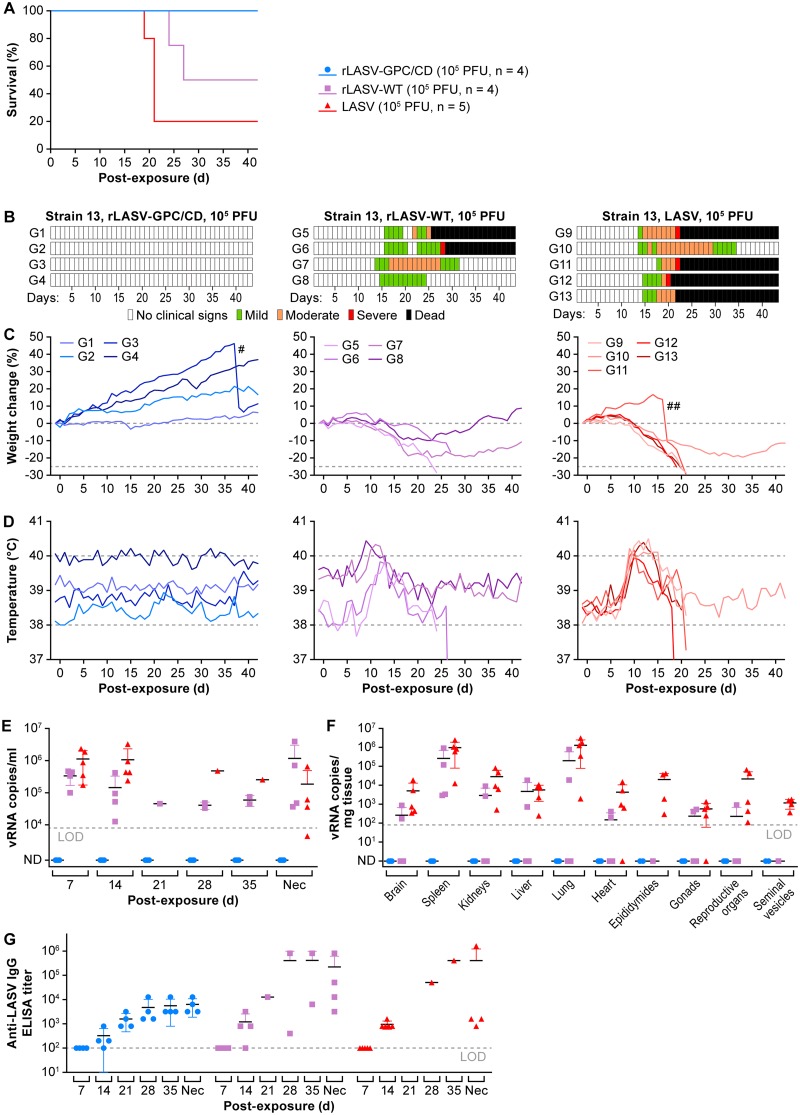
rLASV-GPC/CD is attenuated in strain 13 guinea pigs. Strain 13 guinea pigs were inoculated s.c. with 10^5^ PFU of rLASV-GPC/CD (*n* = 4, blue), rLASV-WT (*n* = 4, purple), or LASV (*n* = 5, red). Survival (A), clinical scores (B), body weight loss (C), and temperature changes (D) were monitored daily for 6 weeks. Viral loads in the blood at different times p.e. (E) and in the indicated tissues at the end of the study (F) were measured by RT-qPCR. Anti-LASV IgG titers were determined by ELISA (G). #, guinea pig G3 gave birth to two healthy pups on day 37 p.e.; ##, guinea pig G11 aborted four fetuses on day 16 p.e.; LOD, limit of detection in each assay; ND, not detected; Nec, necropsy date.

Strain 13 guinea pigs were inoculated s.c. with 10^5^ PFU of rLASV-GPC/CD, rLASV-WT, or LASV. All animals survived inoculation of rLASV-GPC/CD (Fig. 3A) and did not develop any clinical signs of disease (Fig. 3B to D) throughout the duration of the experiment (endpoint, day 42 postexposure [p.e.]). In contrast, all animals inoculated with rLASV-WT or LASV developed several clinical signs of infection (e.g., ruffled scruffy appearance, labored respiratory rate, body weight loss, transient elevated temperatures). By day 27 p.e., two of four guinea pigs inoculated with rLASV-WT and four of five guinea pigs inoculated with LASV reached the euthanasia threshold. Two guinea pigs inoculated with rLASV-WT and one guinea pig inoculated with LASV recovered from infection. In guinea pigs inoculated with rLASV-GPC/CD, vRNA remained undetectable in blood throughout the experiment (Fig. 3E, blue) and in any tissues collected at the experimental endpoint (Fig. 3F). Anti-LASV-IgG plasma titers were detected in rLASV-GPC/CD-inoculated guinea pigs, suggesting they were infected with rLASV-GPC/CD (Fig. 3G). No anti-LASV neutralization antibody titers were detected in rLASV-GPC/CD-inoculated guinea pigs (Supplemental Methods S1) at the end of study (data not shown). In addition, no significant histopathological findings nor LASV NP antigen could be detected by immunohistochemical staining (IHC) in any examined organs and tissues from rLASV-GPC/CD-inoculated guinea pigs euthanized at day 42 p.e. (end of study; Fig. S3 and Supplemental Methods S1). These results demonstrate the complete attenuation of rLASV-GPC/CD in strain 13 guinea pigs.

10.1128/mBio.00039-20.4 FIG S3 Histopathology and immunohistochemical staining of rLASV-GPC/CD-, rLASV-WT-, and LASV-inoculated strain 13 guinea pigs. (A) Lung tissue section. (B) Mesenteric artery section. Black arrows demonstrate periarteritis. Upper panels, hematoxylin and eosin staining; lower panels, IHC staining with anti-LASV-NP MAb. The brown color shows positive cytoplasmic staining of LASV NP. Download FIG S3, TIF file, 2.8 MB. Copyright © 2020 Cai et al. 2020 Cai et al. This content is distributed under the terms of the Creative Commons Attribution 4.0 International license.

In contrast, in guinea pigs inoculated with rLASV-WT or LASV, high concentrations of vRNAs were detected in blood at days 7 and 14 p.e. (average of 10^5^ to 10^6^ LASV vRNA copies/ml) (Fig. 3E). vRNA was also detected in most tissues tested from rLASV-WT or LASV-inoculated guinea pigs (Fig. 3F). All guinea pigs that succumbed to rLASV-WT or LASV had typical acute LF lesions, including interstitial pneumonia (see Fig. S3 and Text S1 in the supplemental material), hepatic degeneration and necrosis (data not shown), endocarditis (data not shown), and splenic lymphoid depletion (data not shown). LASV antigen staining was positive mainly in macrophages, epithelial cells, and/or arterial endothelial cells in most examined organs (Fig. S3). Consistent with previous findings (49), all guinea pigs that survived rLASV-WT or LASV infection had mild to severe systemic, lymphoplasmacytic, and histiocytic periarteritis, with positive LASV antigen staining in the smooth muscle cells in the tunicae media of large arteries (Fig. S3B).

10.1128/mBio.00039-20.1 TEXT S1 Supplemental text and discussion. Download Text S1, DOCX file, 0.04 MB. Copyright © 2020 Cai et al. 2020 Cai et al. This content is distributed under the terms of the Creative Commons Attribution 4.0 International license.

### Strain 13 guinea pigs immunized with rLASV-GPC/CD are protected against lethal LASV infection.

**FIG 4:**
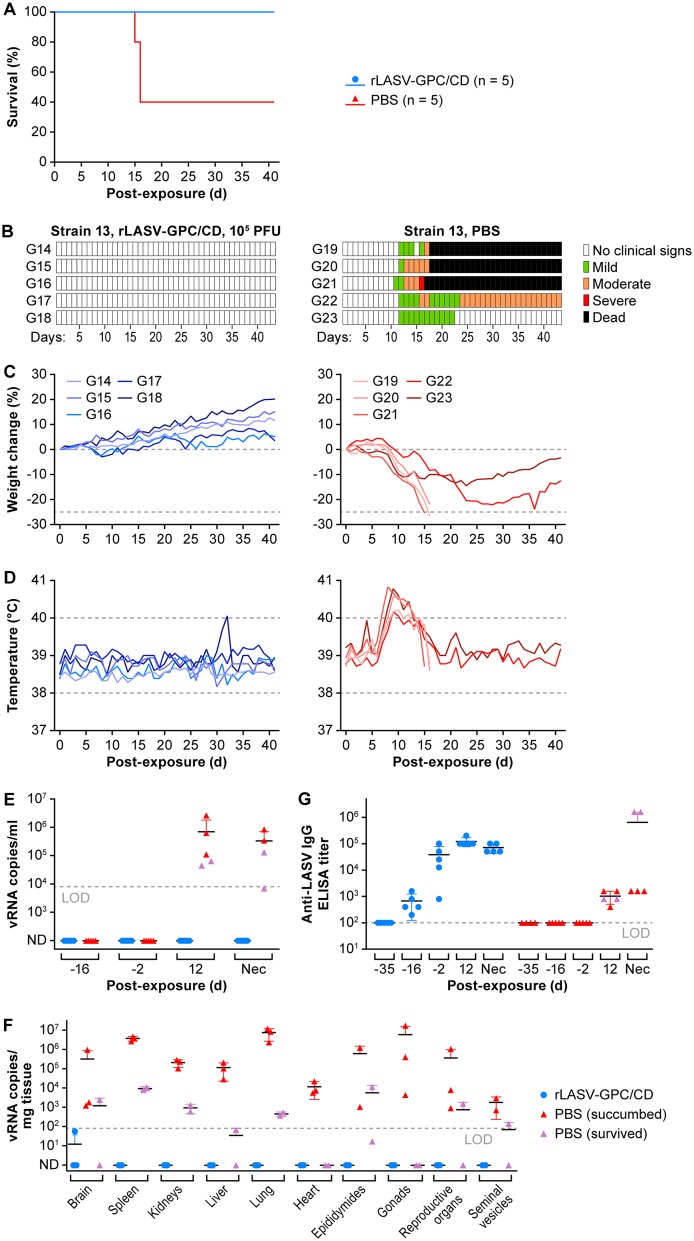
Strain 13 guinea pigs immunized with rLASV-GPC/CD are protected against lethal LASV infection. Strain 13 guinea pigs were immunized s.c. with rLASV-GPC/CD (*n* = 5) or mock-immunized with PBS (*n* = 5). At day 30 p.i., guinea pigs were exposed s.c. to LASV and monitored daily for 6 weeks. Survival (A), clinical scores (B), body weight loss (C), and body temperature changes (D) postexposure are indicated. Viral loads in the blood at different times pre- and postexposure (E) and in the indicated tissues at the time of euthanasia (F) were measured by RT-qPCR. Anti-LASV IgG titers were determined by ELISA (G). LOD, limit of detection in each assay; ND, not detected; Nec, necropsy date.

To test whether a single dose of rLASV-GPC/CD could provide protection from otherwise lethal LASV exposure, strain 13 guinea pigs were immunized s.c. with 10^5^ PFU of rLASV-GPC/CD (*n* = 5) or mock immunized with PBS (*n* = 5, Fig. 4). After 30 days postimmunization (p.i.), guinea pigs were exposed s.c. to 10^5^ PFU of LASV and monitored daily for clinical signs of infection throughout the study. Consistent with previous findings (Fig. 3), administration of rLASV-GPC/CD did not cause any clinical signs (data not shown). All immunized guinea pigs survived LASV exposure (Fig. 4A). In contrast, all mock-immunized guinea pigs lost weight and had elevated temperatures (Fig. 4A to D) after LASV exposure. Three of five guinea pigs from the PBS group succumbed to infection 15 to 16 days post-LASV exposure, one guinea pig developed moderate clinical signs until the study endpoint, and one guinea pig had mild clinical signs and recovered from infection.

On days –16 and –2 pre-LASV exposure (days 14 and 28 after rLASV-GPC/CD immunization), no viremia was detected in any of the rLASV-GPC/CD-immunized animals by reverse transcription-quantitative PCR (RT-qPCR) (Fig. 4E and Supplemental Methods S1). At day 12 p.e. to LASV, vRNA was detected in the blood of all mock-immunized animals (average of 10^6^ LASV vRNA copies/ml) but not in the blood of animals that were previously immunized with rLASV-GPC/CD (Fig. 4E). No vRNA was detected in the tissues tested from rLASV-GPC/CD-immunized animals (Fig. 4F). No significant histopathological lesions nor tissue LASV antigen were detected in rLASV-GPC/CD-immunized guinea pigs (data not shown). Anti-LASV IgG plasma titers were detected in rLASV-GPC/CD immunized guinea pigs 2 weeks p.i. (day –16 pre-LASV exposure) and continued to increase after 4 weeks p.i. (day –2 pre-LASV exposure) (Fig. 4G). However, anti-LASV IgG antibody titers were not significantly boosted after LASV exposure. Interestingly, anti-LASV neutralization titers were not detected in the sera of rLASV-GPC/CD-immunized guinea pigs (Supplemental Methods S1), suggesting that neutralizing antibodies did not play a significant role in the protection conferred by rLASV-GPC/CD (data not shown). Altogether, these data demonstrate that a single inoculation of rLASV-GPC/CD completely protected strain 13 guinea pigs from LASV infection and disease.

### Hartley guinea pigs immunized with rLASV-GPC/CD are protected against lethal LASV infection.

**FIG 5:**
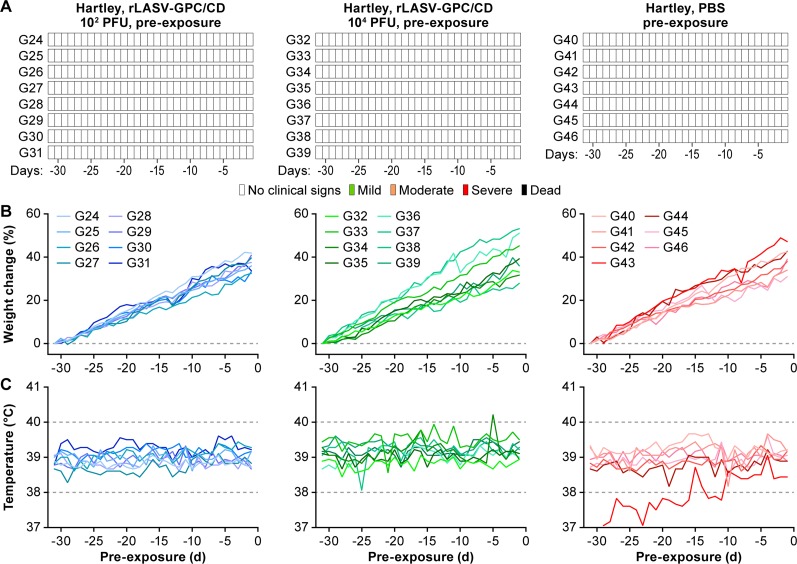
rLASV-GPC/CD is attenuated in Hartley guinea pigs. Hartley guinea pigs were inoculated s.c. with 10^2^ or 10^4^ PFU of rLASV-GPC/CD (*n* = 8) or mock immunized with PBS (*n* = 7). Clinical scores (A), changes in body weight (B), and temperature (C) were monitored daily for 30 days.

Although Hartley guinea pigs are more resistant than strain 13 guinea pig to LASV infection, inoculation of Hartley guinea pigs with 10^4^ PFU intraperitoneally (i.p.) with GPA-LASV results in ≥80% lethality (50; unpublished data). Thus, these guinea pigs are an attractive model for testing the efficacy of LASV candidates due to the limited availability of strain 13 guinea pigs. To evaluate whether a low dose of rLASV-GPC/CD was able to confer protection of Hartley guinea pigs against a lethal GPA-LASV exposure, three groups were immunized s.c. with 10^2^ PFU (low dose, *n* = 8), 10^4^ PFU (high dose, *n* = 8) of rLASV-GPC/CD, or with phosphate-buffered saline (PBS; *n* = 7). Immunization with rLASV-GPC/CD did not cause any clinical signs of disease (Fig. 5), demonstrating the attenuation and safety of rLASV-GPC/CD in Hartley guinea pigs.

**FIG 6:**
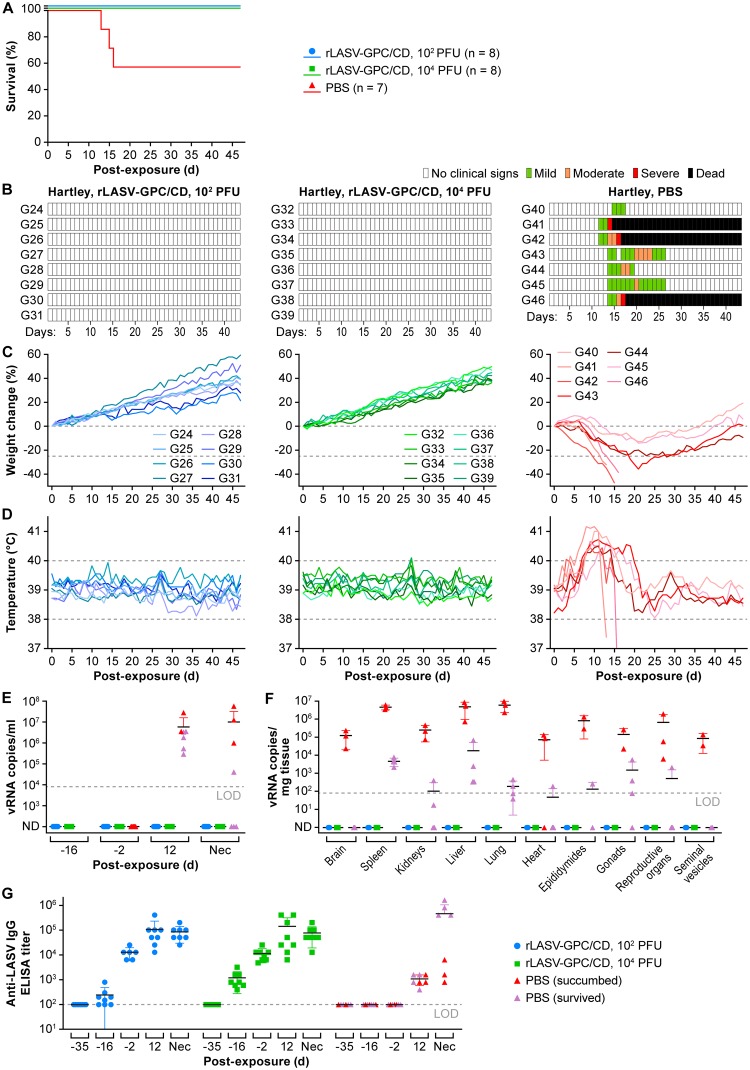
Hartley guinea pigs immunized with rLASV-GPC/CD are protected against lethal LASV infection. Hartley guinea pigs were immunized s.c. with 10^2^ or 10^4^ PFU of rLASV-GPC/CD or mock immunized with PBS (from Fig. 5). At day 30 p.i., guinea pigs were exposed i.p. to 10^4^ PFU of GPA-LASV and monitored daily for 47 days for survival (A), clinical score (B), body weight (C), and body temperature changes (D). Viral loads in the blood at different times pre- and postexposure (E) and in the indicated tissues at the time of euthanasia (F) were measured by RT-qPCR. (G) Anti-LASV IgG titers were determined by ELISA. LOD, limit of detection in each assay. ND, not detected; Nec, necropsy date.

At 30 days p.i., all guinea pigs were exposed i.p. to 10^4^ PFU of GPA-LASV and monitored daily for clinical signs of infection, including body weight, and temperature changes throughout the study (day –31 preexposure to day 47 p.e. with GPA-LASV). Protection against GPA-LASV-associated disease was observed in all guinea pigs immunized with either 10^2^ (*P = *0.0442, log-rank test) or 10^4^ (*P = *0.0442) PFU of rLASV-GPC/CD (Fig. 6A). All immunized guinea pigs survived GPA-LASV exposure without having any clinical signs of disease (Fig. 6B to D). In contrast, all mock-immunized guinea pigs had clinical signs of disease. Three of seven guinea pigs from the control group succumbed to GPA-LASV infection 13 to 16 days p.e., whereas the other four animals recovered from infection.

Viremia was assessed by RT-qPCR on days –16 and –2 preexposure (days 14 and 28 p.i. with rLASV-GPC/CD); no vRNA was detected in any of the rLASV-GPC/CD-immunized Hartley guinea pigs (Fig. 6E and Supplemental Methods S1). Importantly, at day 12 p.e. with GPA-LASV, vRNA was detected in the blood of all mock-immunized guinea pigs (average of 10^7^ vRNA copies/ml) but not in the blood of rLASV-GPC/CD-immunized animals (Fig. 6E). Likewise, in the immunization control group, the vRNA loads were high in most of the tissues collected from animals that succumbed to GPA-LASV infection (Fig. 6F, red). vRNA was detected in different tissues collected from mock-immunized animals that recovered from GPA-LASV infection (Fig. 6F, purple). However, no vRNA was detected in any of the tissues tested from guinea pigs immunized with rLASV-GPC/CD (Fig. 6F, blue and green). None of the tissues from rLASV-GPC/CD-immunized guinea pigs had any significant histopathological lesions, and all were negative for LASV antigen (data not shown).

Anti-LASV IgG titers were detected in rLASV-GPC/CD-immunized guinea pigs by 2 weeks p.i. (day –16 preexposure) and continued to increase after 4 weeks p.i. (day -2 preexposure). However, anti-LASV IgG antibody titers were not significantly boosted after GPA-LASV exposure (Fig. 6G). Anti-LASV neutralizing antibodies were detected in the serum of only 1 of 16 guinea pigs immunized with rLASV-GPC/CD and exposed to GPA-LASV (Supplemental Methods S1), suggesting that neutralization antibodies do not play an important role in the protection provided by rLASV-GPC/CD (data not shown). Altogether, these data demonstrate that a single inoculation of rLASV-GPC/CD completely protected Hartley guinea pigs from LASV infection and disease.

### rLASV-GPC/CD is genetically stable *in vitro*.

**FIG 7:**
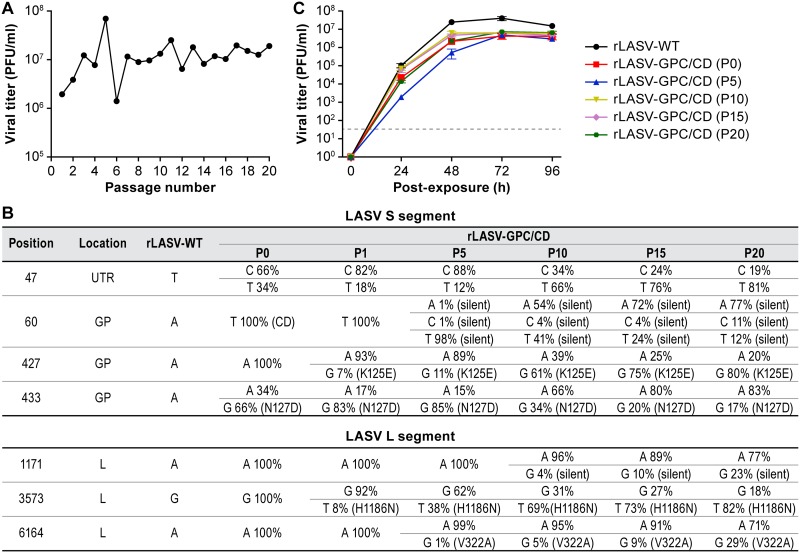
rLASV-GPC/CD is genetically stable *in vitro*. (A) Viral titers of rLASV-GPC/CD at different passages. Vero cells were inoculated (MOI = 0.01) with rLASV-GPC/CD. At 72 h p.e., TCS were collected (P1), and virus titers were determined by plaque assay. Fresh Vero cells were inoculated with TCS from P1 (MOI = 0.01). The process was repeated through P20. (B) Nucleotide and amino acid residue changes of rLASV-GPC/CD after serial passages in Vero cells. vRNA from P1, P5, P10, P15, and P20 of rLASV-GPC/CD was extracted and used for NGS. (C) Growth kinetics of P0, P5, P10, P15, and P20 rLASV-GPC/CD in Vero cells (MOI = 0.01). At the indicated times (h) p.e., TSC were collected, and viral titers were determined by plaque assay. Data represent the means ± the SD of triplicate samples.

Genetic stability is an important feature that needs to be considered in the development of any live-attenuated vaccine (LAV). Therefore, we investigated the genetic stability of rLASV-GPC/CD by serial passaging the virus in Vero cells. To that end, Vero cells were inoculated (MOI = 0.01) with rLASV-GPC/CD. At 72 h p.i., TCS were collected (P1), and viral titers were determined by plaque assay. Fresh Vero cells were inoculated (MOI of 0.01) with rLASV-GPC/CD P1, and this process was serially repeated for a total of 20 passages (P20). Endpoint titers of each passage were similar (Fig. 7A). vRNAs from P0, P1, P5, P10, P15, and P20 were extracted and analyzed by next-generation sequencing (NGS). A single nucleotide polymorphism (SNP) in the GPC gene (A427G→K125E) (Fig. 7B) and an SNP in the L gene (G3573T→H1186N) were identified (Fig. 7B). The growth kinetics of rLASV-GPC/CD from P0, P5, P10, P15, and P20 in infected Vero cells were similar, with no statistical difference in viral peak titer (*P* > 0.05), indicating that the identified SNPs did not change viral fitness *in vitro* (Fig. 7C). These results demonstrate the genetic stability of rLASV-GPC/CD up to 20 passages in Vero cells.

## DISCUSSION

Studies involving LF survivors and animals experimentally infected with LASV indicate that virus-specific CD4^+^ and CD8^+^ T-cell responses are the best correlates of recovery and protection (51–53). Neutralizing antibodies, which appear late after infection and at low titers (54), do not contribute to viral control and recovery during acute LASV infection. However, evidence indicates that neutralizing antibodies genetically engineered from B cells of LF survivors can be successfully used for immunotherapy (55–57). These findings, together with prospective epidemiological studies in Western Africa, indicate that an LAV represents the most feasible approach to control LF, since LAVs often induce long-term robust cell-mediated and humoral responses following a single immunization (58). Several LF vaccine platforms based on vaccinia virus (59, 60), vesicular stomatitis Indiana virus (VSIV) (47, 61), Mopeia virus (MOPV) (62), yellow fever virus (63, 64), and reassortant ML29 carrying the L segment from the nonpathogenic MOPV and the S segment from LASV (65–68) have been promising in animal models, including nonhuman primates (NHPs).

The Coalition for Epidemiological Preparedness Innovation (CEPI) is currently supporting the development of five different LASV vaccine platforms: (i) recombinant VSIV expressing LASV GPC (rVSV-LASV/GPC), (ii) recombinant measles virus (rMV)-expressing LASV GP and NP, (iii) chimpanzee adenovirus (ChAdOxl) expressing LASV GPC, (iv) DNA-based immunization, and (v) mRNA-based immunization. However, effective immunization with rVSV-LASV/GPC requires a high dose that might cause significant VSIV-associated side effects (69–71), rMV-LASV did not provide sterile immunity against LASV infection (72), and no documented evidence supports the protective efficacy of either chimpanzee adenovirus-based or mRNA-based LASV vaccine candidates in LF animal models. The limitations of these LASV vaccine candidates underscore the need for exploring additional vaccine platforms to combat LF in Western Africa.

Protein expression of mammalian viruses is influenced by the codon usage bias of the cells they infect, and, thereby, replacement of commonly used codons with nonpreferred codons decreases protein expression and results in viral attenuation (40, 73–76). We previously demonstrated the feasibility of implementing a CD approach for the generation of an attenuated LCMV based on CD of the viral NP and GPC genes (45, 77). Using a correlation between the degree of NP CD and virus viability and virulence, rescue of rLCMV having an NP gene with a level of CD that permitted efficient virus growth in cultured cells but lack of virulence *in vivo* was feasible (77). Unexpectedly, an rLCMV encoding a fully CD GPC exhibited WT-like growth properties in cultured cells, which is important for vaccine production and manufacturing, but was completely attenuated *in vivo*. rLCMV-GPC/CD was able to completely protect against rLCMV-WT infection and disease, which is important for its implementation as an LAV (45). How this attenuated virus grew efficiently in cultured cells despite drastically reduced GP expression and the mechanisms underlying attenuation *in vivo* remain to be determined.

We demonstrate here that a similar CD approach can also be implemented for LASV using rLASV-GPC/CD (Fig. 1; see also Fig. S2 and Supplemental Methods S1). rLASV-GPC/CD exhibits WT-like growth properties in cultured cells despite lower GP expression (Fig. 2). The virus is attenuated in both strain 13 (Fig. 3 and 4) and Hartley (Fig. 5) guinea pigs and is able to confer complete protection with a single low dose against LASV (Fig. 4 and 6). Moreover, we also demonstrate that rLASV-GPC/CD is genetically stable in cultured cells (Fig. 7), another important feature for implementation as a LAV candidate. We identified an SNP (A427G) in the GPC gene that resulted in substitution K125E. Positions K125 and K126 in LASV GPC were suggested to be involved in the interaction of LASV GP1 with its receptor dystroglycan 1 (DAG1) (78). However, LASV enters Vero cells in a DAG1-independent manner. Consequently, LASV GP1 residues involved in binding to DAG1 would not be expected to play a critical role in LASV infection of Vero cells (79, 80). Accordingly, we did not observe an impact of K125E mutation on the growth properties of rLASV-GPC/CD in Vero cells. The impact of the K125E mutation on the growth properties of rLASV-GPC/CD in other cell types, which can only be infected in a DAG1-dependent manner, remains to be investigated. Future studies in NHP animal models will help to demonstrate the feasibility of implementing rLASV-GPC/CD as a LAV for the treatment of human LASV infections.

Compared to other LAV approaches, the CD approach offers a number of advantages. (i) CD relies on the introduction of a large number (hundreds) of synonymous mutations in a viral protein (e.g., LASV GPC; Fig. S1), making reversion to a virulent WT sequence highly unlikely. (ii) CD does not result in amino acid residue changes in the viral protein (Fig. 1); the CD virus retains the same antigenic epitopes as WT virus. (iii) LAV based on CD viruses can be rapidly generated by combining *de novo* gene or genome synthesis and reverse genetics approaches (Fig. S2 and Supplemental Methods S1). (iv) Finally, CD-based attenuation of viruses, including rLASV-GPC/CD, is safe (Fig. 3 and 5) and is able to confer complete protection against WT viruses by using a single immunization dose (Fig. 4 and 6). Our results also suggest that rLASV-GPC/CD might be a safe LASV surrogate to study LASV-host cell interactions outside biosafety level 4 (BSL-4) laboratories.

LAVs represent the most widely and successful approach to protect against human viral diseases, including measles, mumps, rubella, rotavirus infections, and chickenpox, and infections with several high-consequence pathogens, such as variola virus or yellow fever (81). Likewise, Candid#1 is a safe and effective LAV against Argentinian hemorrhagic fever (AHF) caused by the mammarenavirus Junín virus (JUNV). Indeed, Candid#1 has been licensed in Argentina to protect people in areas where JUNV is endemic (82, 83). Most LAVs currently in use to treat human viral diseases were developed by serial virus passages in animals or cell substrates, or a combination of both, during which mutations fixed in the viral genome resulted in attenuation, as determined by testing in appropriate animal models. LAVs offer the key advantage of mimicking the course of WT virus natural infections, which often results in long-lasting protective immunity, including both humoral and T cell responses. However, a key concern of LAVs is the potential for the genetic reversion of critical attenuating mutations or adaptation to host-specific susceptibility factors that could result in increased virus virulence. In addition, the unequivocal identification of the genetic determinants of viral attenuation may not result in the elucidation of mechanisms of attenuation. The rLASV-GPC/CD LAV candidate overcomes these problems as the incorporation of 379 nucleotide mutations within the GPC gene, affecting 321 of 491 codons, poses an insurmountable genetic barrier for the generation of revertant viruses with increased virulence. Future studies aimed at developing rLASV-GPC/CD as an LF vaccine for human use will involve testing its safety and protective efficacy, including the lowest protective dose, in NHPs. Safety studies should both assess whether rLASV-GPC/CD can cause sensorineural hearing loss, which is often observed in LF survivors, and the vaccine’s safety in immunocompromised NHPs. The target population may include individuals with different degrees of immune suppression due to the prevalence of HIV-1 and plasmodium infections in Western Africa.

## MATERIALS AND METHODS

### Cell lines.

Human adenocarcinoma alveolar basal epithelial A549 (American Type Culture Collection [ATCC], Manassas, VA, USA; CCL-185), human embryonic kidney epithelial (HEK293T/17) (ATCC, CRL-11268), grivet (Cercopithecidae: Chlorocebus aethiops Linnaeus, 1758), kidney epithelial Vero (ATCC, CCL-81), and Vero E6 (BEI Resources, Manassas, VA, USA; NR596) cells were grown in Gibco Dulbecco modified Eagle medium (Thermo Fisher Scientific, Waltham, MA) supplemented with 10% heat-inactivated fetal bovine serum (Sigma-Aldrich, St. Louis, MO). All cells were incubated at 37°C in a humidified 5% CO_2_ atmosphere.

### Viruses.

LASV isolate Josiah and guinea pig-adapted LASV isolate Josiah (GPA-LASV) (84) were provided by the U.S. Army Medical Research Institute of Infectious Diseases (Fort Detrick, Frederick, MD). LASV, GPA-LASV (stock IRF0205; L segment GenBank KY425651.1; S segment GenBank KY425643.1) (50), and recombinant Josiah isolate-based virus (rLASV-WT, rLASV-GFP, and rLASV-GPC/CD) stocks were prepared in Vero or Vero E6 cells using MOIs of 0.01. At day 3 postexposure (p.e.), tissue culture supernatants (TCS) were collected and clarified by centrifugation at 7,500 × *g* for 10 min, aliquoted, and stored at –80°C until use. Virus titers were determined by plaque assay using Vero cells as described previously (85).

### Plasmids.

Plasmids were generated as outlined in Supplemental Methods S1.

### Western blot analysis.

In transfection experiments, HEK293T/17 cells were transiently transfected using LPF2000 with 1 μg of empty or WT or CD LASV GPC pCAGGS expression plasmids. Transfected cells were collected and lysed at 48 h p.t. In infection experiments, A549 or HEK293T/17 cells were inoculated with rLASV-WT or rLASV-GPC/CD at an MOI of 0.1. At various times p.i., cell monolayers were lysed using cell lysis buffer (Cell Signaling Technology, Danvers, MA). Lysates were gamma irradiated and transferred from the BSL-4 to a BSL-2 laboratory.

Equivalent amounts of total cell lysates (20 μg) were separated on 4 to 12% Bis-Tris NuPAGE gels (Thermo Fisher Scientific) and then dry transferred onto nitrocellulose membranes using the iBlot 2 gel transfer system (Thermo Fisher Scientific). In transfection experiments, membranes were incubated with the cross-reactive anti-LCMV GPC/GP2 monoclonal antibody (MAb) 83.6 (28, 86) or anti-glyceraldehyde-3-phosphate dehydrogenase (GAPDH) antibody (Abcam, Cambridge, MA). In infection experiments, membranes were incubated with anti-LASV GP2 polyclonal antibody (0307-001; IBT Bioservices, Rockville, MD), anti-LASV NP MAb (01-0400104; Cambridge Biologics, Brookline, MA), or anti-actin beta antibody (ab8227; Abcam). In both experiments, membranes were incubated with horseradish peroxidase (HRP)-conjugated secondary antibodies (Sigma-Aldrich). Signals were detected using SuperSignal West Femto maximum sensitivity substrate (Thermo Fisher Scientific), and the images were acquired with a G:BOX chemiluminescence imaging system (Syngene, Frederick, MD). Band densities were analyzed by ImageJ software (National Institutes of Health).

### Immunofluorescence assays.

Transfection experiments: HEK293T/17 cells were transiently transfected using LPF2000 with 1 μg of empty or WT or CD LASV GPC pCAGGS expression plasmids. At 48 h p.t., the cells were fixed with 4% paraformaldehyde (Electron Microscopy Sciences, Hatfield, PA) and then stained with MAb 83.6, followed by fluorescein isothiocyanate-conjugated rabbit anti-mouse IgG antibody (Dako, Carpinteria, CA). Protein expression was visualized under a Leica fluorescence microscope (Leica Microsystems, Buffalo Grove, IL). Images were colored using Adobe Photoshop CS4 (v11.0) software (Adobe, San Jose, CA). LASV GPC expression in the immunofluorescence assay was quantified by using ImageJ. The relative intensity of rLASV-GPC/CD was calculated by normalizing to the levels of LASV GPC WT expression.

For the infection experiments, A549 or Vero cells (3 × 10^4^ cells/well, 96-well plate format) were inoculated with rLASV-GPC/CD or rLASV-WT at MOIs of 0.01 or 0.1. At various times p.e., the cells were fixed with 10% neutral buffered formalin (Thermo Fisher Scientific) for 24 h and then stained with human anti-LASV GP MAb 37.2G (56), followed by secondary Alexa Fluor 594-conjugated goat anti-human IgG antibody (Life Technologies, Carlsbad, CA) or by mouse anti-LASV NP MAb 100LN IgG2b (Autoimmune Technologies, New Orleans, LA), followed by secondary Alexa Fluor 488-conjugated goat anti-mouse IgG antibody (Life Technologies). Hoechst 33342 dye (Thermo Fisher Scientific) was used to stain cell nuclei. Fluorescent signal images were acquired with an Operetta high-content imaging system and analyzed by using Harmony 3.1 software (Perkin-Elmer, Waltham, MA).

### Animal studies.

All animal studies were approved by the Division of Clinical Research (DCR) Institutional Animal Care and Use Committee and performed at the National Institutes of Health, National Institute of Allergy and Infectious Diseases, DCR, Integrated Research Facility (NIH/NIAID/DCR/IRF-Frederick), which is fully accredited by the Association for Assessment and Accreditation of Laboratory Animal Care International (AAALAC). Six to 16-week-old male and female strain 13 guinea pigs (Rodentia: Caviidae: Cavia porcellus Linnaeus, 1758) were obtained from the NIH/NIAID/DCR/IRF-Frederick breeding colony. Six-week-old male and female Hartley guinea pigs were obtained from Charles River Laboratories, Wilmington, MA. Guinea pigs were housed in an animal BSL-4 laboratory in high-efficiency particulate air (HEPA)-filtered microisolator cage systems (Lab Products, Seaford, DE). For all animal studies, animals were monitored daily for clinical signs, and daily body weight and temperature readings were obtained throughout. Animals were euthanized once clinical illness scores (e.g., hunching, paralysis, head tilting with rolling, inability to each/drink, eye closure, agonal breathing, tremors, hypothermia) indicated terminal stages of disease or at the completion of study (day 42 or 47 p.e.). Complete necropsies were performed on all animals.

### Safety evaluation of rLASV-GPC/CD in strain 13 guinea pigs.

Groups of four or five guinea pigs, which were not proportionally distributed by age and sex because of limited availability, were exposed s.c. to 10^5^ PFU of rLASV-GPC/CD, rLASV-WT, or LASV. Blood samples were collected from the cranial venae cavae at days 7, 14, 21, 28, 35, and 42 p.e.

### Efficacy evaluation of rLASV-GPC/CD in strain 13 guinea pigs.

Groups of guinea pigs were immunized s.c. with 10^5^ PFU of rLASV-GPC/CD or were mock immunized with PBS. At 30 days p.i., all animals were exposed s.c. to 10^5^ PFU of LASV. Blood samples were collected at day 5 before immunization, on days 14 and 28 p.i., and on day 12 p.e.

### Efficacy evaluation of rLASV-GPC/CD in Hartley guinea pigs.

Animals were divided into three groups of seven or eight animals, distributed proportionally by age and sex. Groups were immunized s.c. with 10^2^ or 10^4^ PFU of rLASV-GPC/CD (*n* = 8) or mock immunized with PBS (*n* = 7). At 30 days p.i., all animals were exposed i.p. to 10^4^ PFU of GPA-LASV. Blood samples were collected at day 5 before immunization, on days 14 and 28 p.i., and on day 12 p.e.

### Reverse transcriptase-quantitative polymerase chain reaction.

Viral loads in whole blood or tissues samples were measured using reverse transcriptase-quantitative PCR (RT-qPCR) (Supplemental Methods S1).

### Virus neutralization assay.

Antibody neutralization titers were measured using a fluorescence-based neutralization assay and a green fluorescent protein-expressing rLASV-GFP (Supplemental Methods S1).

### Endpoint anti-LASV IgG enzyme-linked immunosorbent assay.

LASV antigens for enzyme-linked immunosorbent assay (ELISA) were obtained from crude cell extracts of Vero cells infected with LASV. Cells were harvested and washed with PBS, then lysed in RIPA Buffer (9806S; Cell Signaling Technology) with cOmplete protease inhibitor cocktail tablets (Roche, Indianapolis, IN). The lysates were incubated at 4°C for 10 to 20 min before freezing at −80°C. Cell extracts were gamma irradiated (50 kGy) to inactivate viable virus before the extracts were transferred from the BSL-4 to the BSL-2 laboratory. The irradiated cell lysates were sonicated, followed with clarification by centrifugation at 10,000 × *g* at 4°C for 15 min. The cell extracts were aliquoted and frozen at –80°C. Inactivated cell extracts were diluted with carbonate buffer (pH 9.5; BioLegend, San Diego, CA), coated onto plates at a final protein concentration of 50 ng/well, and incubated at 4°C overnight. Plates were washed six times with PBST (PBS plus 0.2% Tween 20 [Sigma-Aldrich]) before 300 μl of blocking buffer (PBST plus 3% normal chicken serum [Abcam] plus 2% milk [Thermo Fisher Scientific]) was added, followed by incubation at 37°C for 2 h. Twofold serial dilutions of heat-inactivated irradiated test plasma were added to the plates, followed by incubation at 4°C overnight. Plates were washed six times with PBST before the addition of goat anti-guinea pig IgG-HRP (Sigma-Aldrich) and then incubated at 37°C for 1 h. The plates were washed again with PBST and developed with 3,3′,5,5′-tetramethylbenzidine substrate (Thermo Fisher Scientific). The reaction was stopped by addition of stop solution, and the optical density was measured at 450 nm on an Infinite M1000 plate reader (Tecan, Morrisville, NC). Endpoint titers were measured, and reciprocal plasma dilutions corresponding to minimal binding were used to calculate titers. The average signal from normal guinea pig plasma plus 3× standard deviations was set as the cutoff value for endpoint titer measurement (87).

### Histology and immunohistochemical staining.

Necropsies of guinea pigs, tissue collection, and staining were performed by a board-certified veterinary pathologist (Supplemental Methods S1).

### Assessment of rLASV-GPC/CD genetic stability.

To assess genetic stability, rLASV-GPC/CD was passaged 20 times in Vero cells. Briefly, Vero cells were inoculated with rLASV-GPC/CD (defined as passage 0 [P0]) at an MOI of 0.01. At 72 h p.i., TCS were collected (P1), and virus titers were measured by plaque assay. Fresh Vero cells were then inoculated with P1 at an MOI of 0.01 to generate P2. This process was serially repeated until P20. P0, P1, P5, P10, P15, and P20 samples of rLASV-GPC/CD were resuspended in TRIzol LS, and vRNA was extracted using a Purelink RNA minikit (Thermo Fisher Scientific) according to the manufacturer’s instructions. Purified vRNA (500 ng) was sent to the University of Rochester Genomic Research Center for next-generation sequencing (NGS) analysis. NGS data were aligned with the genomic sequences of rLASV-WT (IRF0297, L segment, GenBank accession no. MH358389; S segment, GenBank accession no. MH358388) using Integrative Genomic Viewer (IGV 2.4.18; Broad Institute, Cambridge, MA) (88), and the percentage of mutations was calculated based on the allele read counts.

### Statistical analysis.

GraphPad Prism 7 was used for all statistical analyses. A log-rank (Mantel-Cox) test was used for survival curve comparison. Statistically significant differences in plaque size and viral titer were determined by unpaired Student *t* test (*, *P < *0.05, significant; **, *P < *0.01, very significant; ***, *P < *0.001, highly significant; ns, *P > *0.05, not significant).

### Data availability.

We declare that all relevant data are available from the corresponding author upon request.
