# Supplementary figures and images for: A Lassa Fever Live-Attenuated Vaccine Based on Codon Deoptimization of the Viral Glycoprotein Gene
Source: mBio. 2020 Feb 25;11(1):e00039-20. doi: 10.1128/mBio.00039-20 (PMC7042690; doi:10.1128/mBio.00039-20)

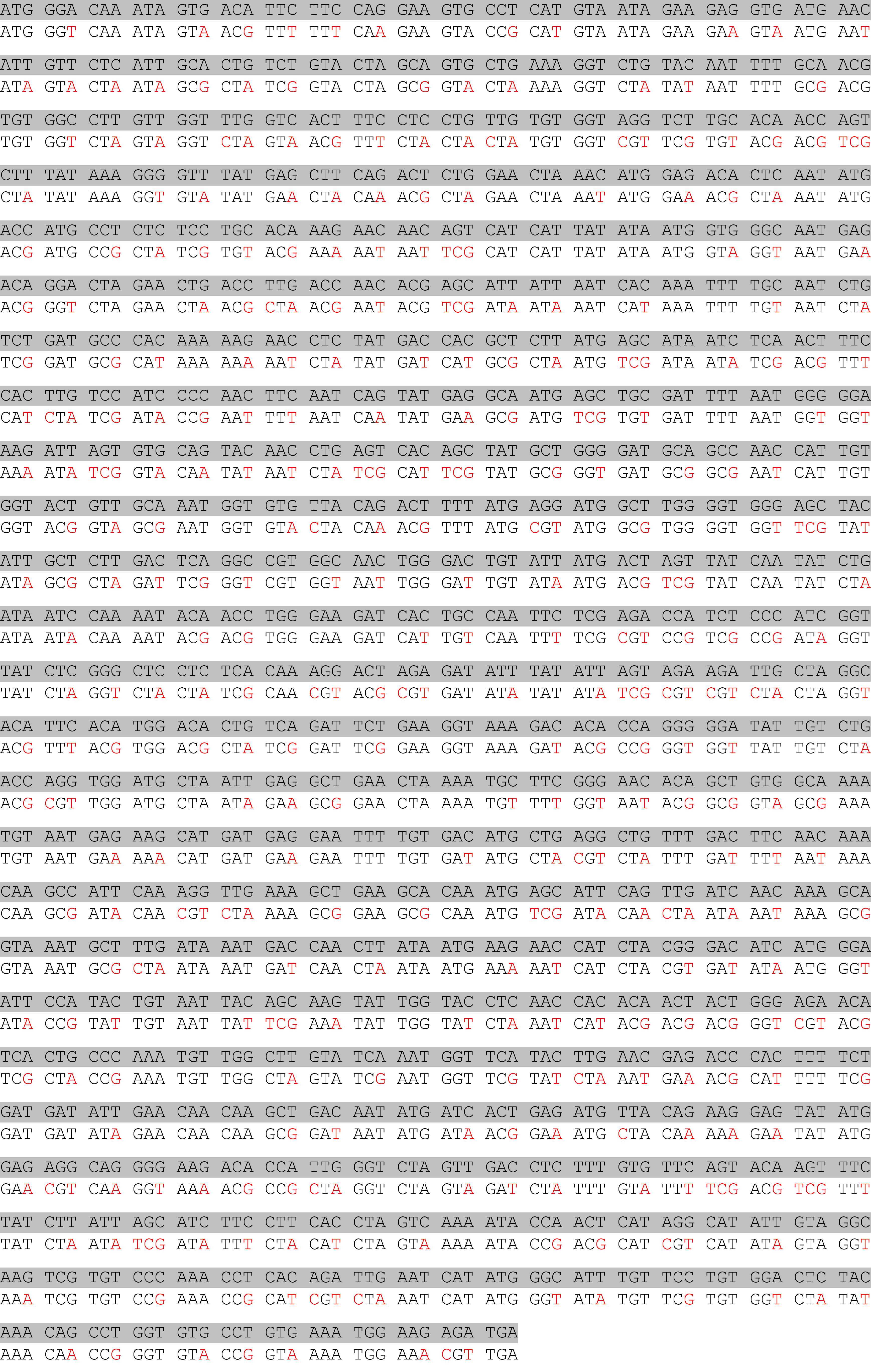

Supplement: FIG S1 [file mBio.00039-20-sf001.tif]

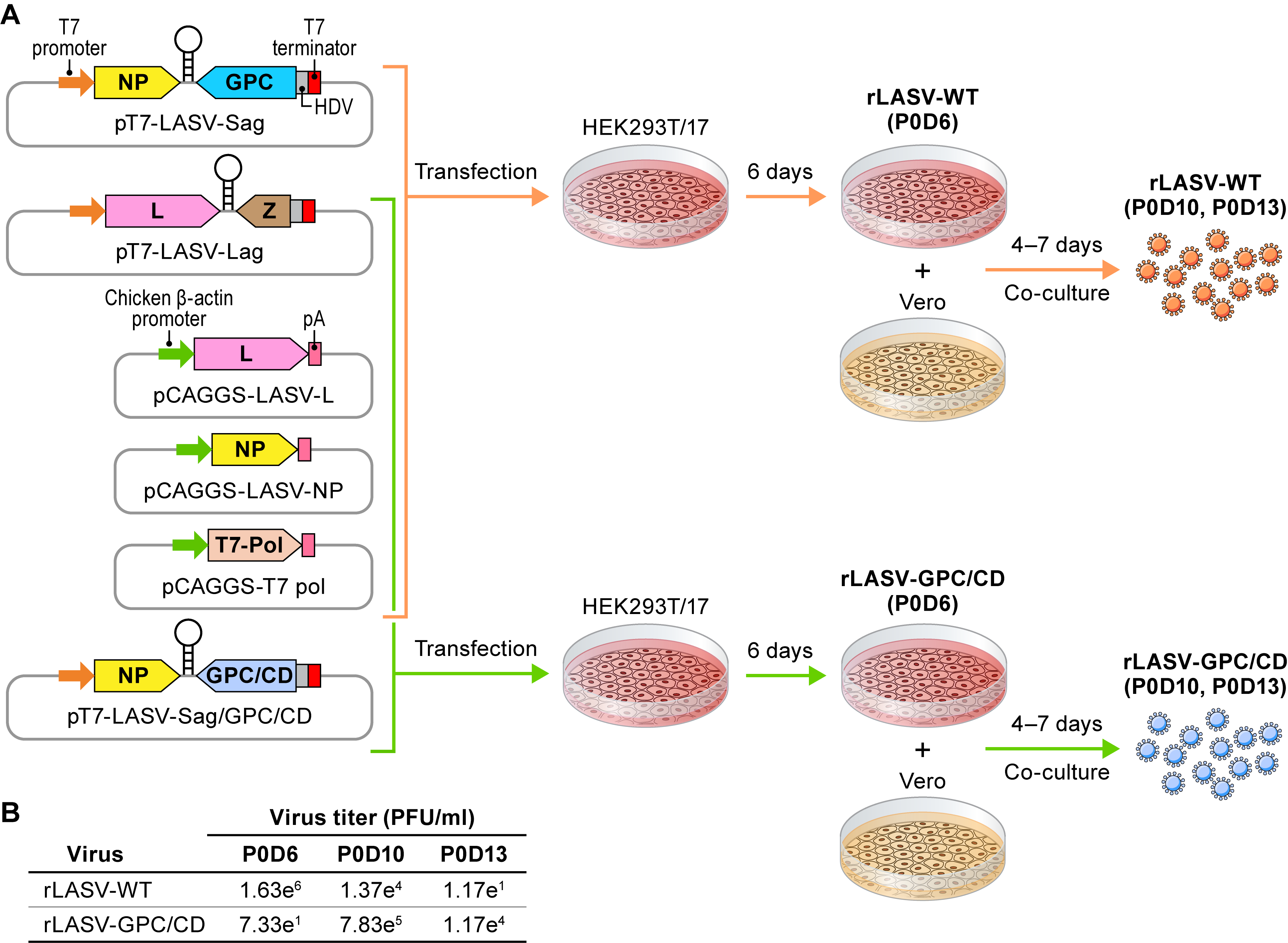

Supplement: FIG S2 [file mBio.00039-20-sf002.tif]

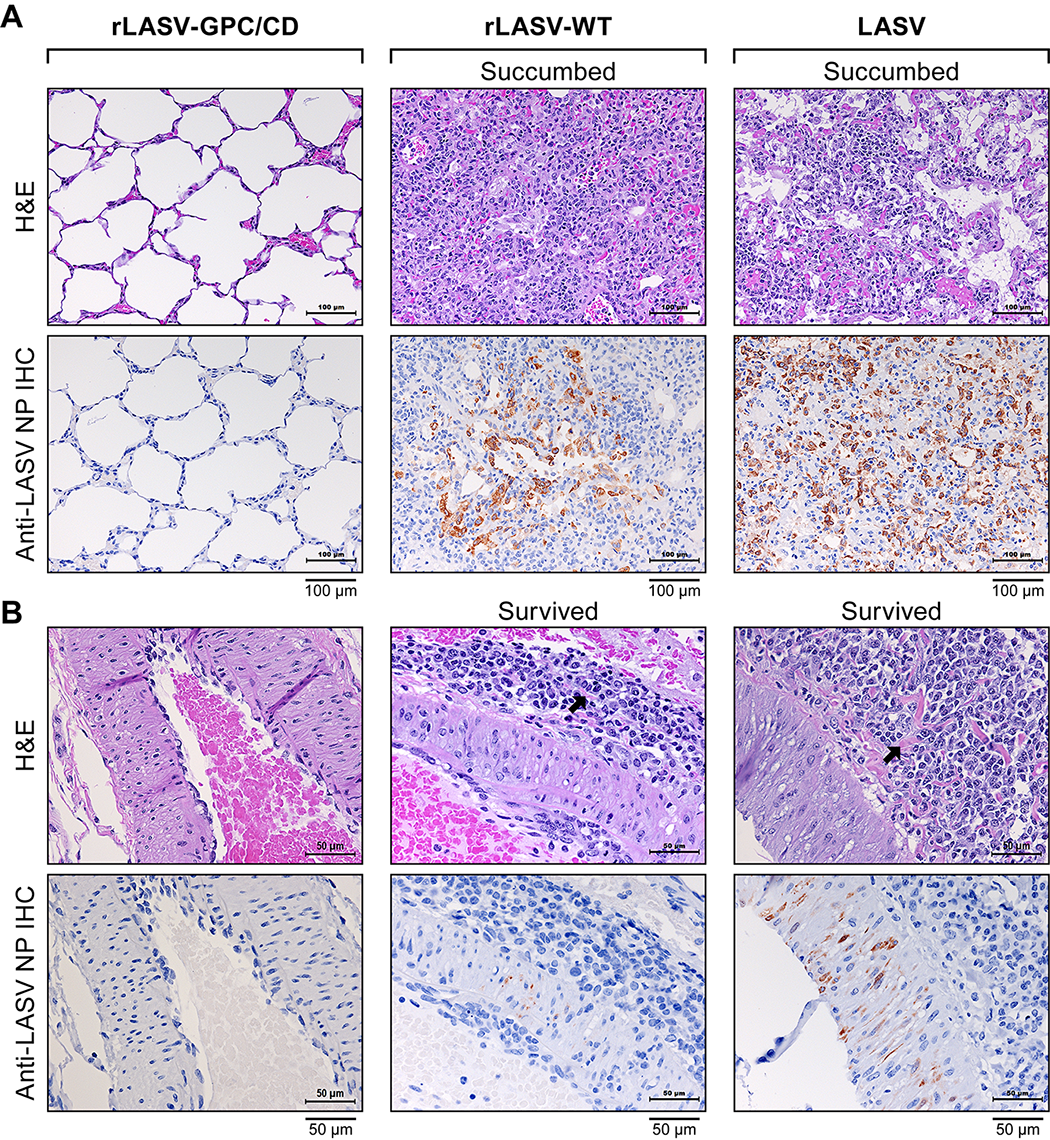

Supplement: FIG S3 [file mBio.00039-20-sf003.tif]
