# Supplementary material for: A Lassa Fever Live-Attenuated Vaccine Based on Codon Deoptimization of the Viral Glycoprotein Gene
Source: mBio. 2020 Feb 25;11(1):e00039-20. doi: 10.1128/mBio.00039-20 (PMC7042690; doi:10.1128/mBio.00039-20)
Supplement: TEXT S1 [file mBio.00039-20-s0001.docx]

**Text S1. Supplemental Materials and Methods**

**Contents of Text S1**

1. Supplemental Material and Methods
2. Plasmids
3. Rescue and propagation of rLASV-GPC/CD and rLASV-WT
4. Reverse transcriptase quantitative polymerase chain reaction
5. Virus neutralization assays
6. Histopathology and immunohistochemical staining
7. References for Text S1
8. **Supplemental Materials and Methods**
9. **Plasmids**

A complete CD LASV GPC gene, in which WT codons were replaced with the least representative codons in mammalian cells (**Fig. S1, Supplemental material**), was generated by *de novo* synthesis and cloned into the pUC57 vector (Biomatik, Wilmington, DE, USA) to generate pUC57-LASV-GPC/CD. To generate pCAGGS-LASV-GPC/CD, the LASV-GPC/CD fragment, flanked by *Eco*RI and *Xho*I restriction sites, was amplified from pUC57-LASV-GPC/CD by PCR and subcloned into the pCAGGS plasmid using standard molecular biology techniques (primer sequences available upon request). pCAGGS plasmids encoding the WT LASV GPC (pCAGGS-LASV-GPC), LASV-L (PCAGGS LASV-L), LASV-NP (pCAGGS LASV-NP), or T7 polymerase (pCAGGS T7) were described previously ([1-3](#_ENREF_1)). To generate mPol-I/LASV-Sag/GPC/CD, pUC57-LASV-GPC/CD was digested with *Bsm*BI, and the generated LASV-GPC/CD fragment was used to replace the GPC WT ORF in plasmid mPol -I/LASV-Sag, the LASV S RNA segment-encoding plasmid ([1](#_ENREF_1)).

To generate plasmid pT7-LASV-Sag, able to direct T7 RNA polymerase (T7pol)-mediated synthesis of full-length LASV S genome RNA, we digested mPol-I-LASV-Sag ([1](#_ENREF_1)) with *Avr*II. Subsequently, the generated DNA fragment encoding the LASV 3′-untranslated region (UTR), NP, IGR, GPC, and 5′-UTR was cloned into a T7 pol-based LCMV S genome RNA expressing plasmid (pT7-S(+)HR) ([4](#_ENREF_4)). This plasmid was digested with *Avr*ll to remove the LCMV S genome segment. We used the same strategy to generate pT7-LASV-Sag/GPC/CD based on mPol-I/LASV-Sag/GPC/CD.

To generate plasmid pT7-LASV-Lag, able to direct T7pol-mediated synthesis of full-length LASV L genome RNA, we generated an intermediate construct by digesting plasmid mPol-I-LASV-L (+), which encodes the *cis*-acting regulatory sequence for the LASV L genome segment and matrix protein (Z) gene but lacks the L gene, with *Avr*II. The *Avr*II DNA fragment encoding 3′-UTR, a linker sequence, IGR, Z, and 5′-UTR was subcloned into a T7pol-based LCMV L genome RNA expressing plasmid (pT7-L(+)HDR) ([4](#_ENREF_4)), resulting in generation of pT7-LASV-L(+)∆L. We generated via PCR a DNA fragment that encoded a region from *Bbs*I site in 3′-UTR of the LASV L segment to *Bst*BI site in Z gene and that lacked a region between *Bps*EI and *Nsi*I sites in the L gene connected with a linker sequence. We digested this PCR product with *Bbs*I and *Bst*BI, and subcloned the resulting fragment into pT7-LASV-L (+) ∆L to generate pT7-LASV-L (+) ∆*Bps*EI-*Nsi*I. We generated via PCR a DNA fragment that encoded a region from *Bps*EI site to *Bsr*GI site of L gene and that lacked a region between *Bsr*GI and *Nsi*I sites connected with a linker sequence and digested the PCR product with *Bps*EI and *Nsi*I. We subcloned the *Bps*EI-*Nsi*I fragment into pT7-LASV-L (+) ∆*Bps*EI-*Nsi*I to generate pT7-LASV-L (+) ∆*Bsr*GI-*Nsi*I. Finally, we generated via PCR a DNA fragment encoding a region from *Bsr*GI site to *Nsi*I site of the L gene that was amplified and digested the PCR product with *Bps*EI and *Nsi*I. We subcloned the *Bsr*GI-*Nsi*I fragment into pT7-LASV-L (+) ∆*Bsr*GI-*Nsi*I, to generate pT7-LASV-Lag. Plasmid constructs were verified by DNA sequencing (ACGT Inc., Wheeling, IL, USA). All restriction enzymes were purchased from New England Biolabs (NEB; Ipswich, MA, USA).

1. **Rescue and propagation of rLASV-GPC/CD and rLASV-WT**

All experiments resulting in or with LASV were performed under maximum (biosafety level 4 [BSL-4]) containment at the National Institutes of Health/National Institute of Allergy and Infectious Diseases/Division of Clinical Research/Integrated Research Facility at Fort Detrick (NIH/NIAID/DCR/IRF-Frederick) following approved standard operating procedures. HEK293T/17 cells (7 x 10^5^ cells/well, 6-well plate format) were co-transfected with 0.6 µg of pCAGGS LASV-NP, 1.0 µg of pCAGGS LASV-L, 1.0 µg of pCAGGS T7, 1.2 µg of pT7-LASV-Lag, and 0.6 µg of pT7-LASV-Sag (rLASV-WT) or pT7-LASV-Sag/GPC-CD (rLASV-GPC/CD), and 11 µl of Lipofectamine 2000 (LPF2000; Thermo Fisher Scientific). At 5 h post-transfection (pt), media were replaced with 3 ml of DMEM containing 2% FBS and 1X Penicillin/Streptomycin (Thermo Fisher Scientific). At 3 days pt, TCS were collected (passage 0, day 3), and 3 ml of fresh media were added to the transfected HEK293T/17 cells. At day 6 pt, TCS were harvested. Transfected HEK293T/17 cells were detached from plates into a suspension of single cells that were then co-cultured with fresh Vero cells (2 x 10^6^ cells). TCS were harvested 4 (day 10 pt) and 7 (day 13 pt) days after co-culture.

Virus titers were determined by plaque assay in Vero cells as previously described ([1](#_ENREF_1)). Briefly, Vero cells (2.5 x 10^5^ cells/well, 6-well plate format) were infected with 10-fold serial dilutions of LASV. After 1 h of incubation at 37ºC, 2 ml of a primary overlay of agarose (Lonza, Rockland, ME, USA) were added to each well. At day 3 PE with rLASV-WT or 4 days PE with rLASV-GPC/CD, 2 ml of a secondary overlay of agarose and 0.03% neutral red stain (EMD Millipore, Burlington, MA, USA) were added to each well. After incubation at 37ºC for 24 h, plaques were counted manually. For plaque morphology visualization, agarose overlays were removed, and plates were stained with 0.2% crystal violet (Ricca Chemical Company, Arlington, TX, USA). Plaque size was measured from 25 randomly selected plaques using Image J software (NIH, Bethesda, MD, USA).

1. **Reverse transcriptase quantitative polymerase chain reaction**

Viral loads in whole blood or tissues samples were measured using reverse transcriptase-quantitative polymerase chain reaction (RT-qPCR) as previously described ([5](#_ENREF_5)). Whole blood or tissue homogenate samples were inactivated by TRIzol LS (Thermo Fisher Scientific). Total RNA was isolated using the Viral RNA Mini Kit (Qiagen, Germantown, MD, USA). Briefly, 70 µl of TRIzol LS-inactivated sample were added to 280 µl of Buffer AVL containing carrier RNA. After the binding and washing steps, the sample was eluted in 70 µl of Buffer AVE. The QuantiFast Pathogen RT-PCR Kit (Qiagen) was used for RT-qPCR. In the 25-µl-RT-qPCR reaction mixture, 5 µl of RNA samples were mixed with 5 µl of 5X master mix, 0.25 µl of QuantiFast Pathogen RT Mix, 0.25 µl of 100 µM of forward primer (5′-CCACCATYTTRTGCATRTGCCA-3′), 0.25 µl of 100 µm of reverse primer (5′-GCACATGTNTCHTAYAGYATGGAYCA-3′), 0.05 µl of 100 µM probe (5′-FAM-AARTGGGGYCCDATGATGTGYCCWTT-BBQ-3′), 0.5 µl of 50X ROX Dye Solution, and 13.7 µl of distilled water (dH_2_O). RT-qPCR was performed at 50°C for 20 min for reverse transcription, 95°C for 5 min to inactivate reverse transcriptase, 45 cycles of 95°C for 15 s for PCR amplification, and 60°C for 30 s on an ABI 7500 real-time PCR system (Applied Biosystem, Foster City, CA, USA). The standard curve spanned 10^8^ copies/reaction (upper limit of quantification, ULOQ) through 10 copies/reaction (lower limit of quantification, LLOQ). Transformed data from whole blood and tissue samples were plotted in viral RNA copies (log_10_)/ml and viral RNA copies (log_10_)/mg tissue, respectively.

1. **Virus neutralization assays**

Antibody neutralization titers were measured using a fluorescent-based neutralization assay and a green-fluorescent protein-expressing rLASV (rLASV-GFP) as previously described ([1](#_ENREF_1)). Briefly, heat-inactivated guinea pig serum samples were two-fold serially diluted (starting dilution of 1:10) and incubated with a fixed amount of rLASV-GFP (3,000 PFU/well) for 1 h at 37°C. Then the virion-antibody mixtures were added onto A549 cell monolayers (3x10^4^ cells/well, 96 well plates). After 48 h incubation at 37°C, plates were fixed with 10% NBF for 24 h and then transferred from the BSL-4 laboratory to the BSL-2 laboratory. GFP signal was measured by an Infinite M100 plate reader (Tecan). Non-linear regression analysis and curve fitting parameters (variable slope) were performed to calculate the reciprocal serum dilutions corresponding to 50% inhibitory concentrations (IC_50_) using Prism GraphPad 7 (GraphPad Software, La Jolla, CA, USA).

1. **Histopathology and immunohistochemical staining**

Necropsies were performed on each guinea pig by a board-certified veterinary pathologist. Tissues were collected and fixed in 10% NBF for at least 72 h before transfer from the BSL-4 to the BSL-2 laboratory. Tissues were then embedded in paraffin, sectioned, mounted on glass slides, and stained with hematoxylin and eosin (H&E) following standard procedures. LASV immunohistochemistry (IHC) staining was performed on routinely processed tissue slides with an anti-LASV-NP mAb (Cat #: 01-04-0104; Cambridge Biologics), sequentially followed by a biotinylated anti-mouse (Cat #: 111-065-166; Jackson Immunoresearch Laboratories, Westgrove, PA, USA) secondary antibody and an avidin-biotin peroxidase tertiary antibody (Cat #: PK-6100; Vector Laboratories, Burlingame, CA, USA). Slides were examined by a board-certified veterinary pathologist using a BX51 light microscope (Olympus, Melville, NY, USA), and photos were taken using a DP73 camera (Olympus).

1. **References for Text S1**

1. Caì Y, Iwasaki M, Beitzel BF, Yú S, Postnikova EN, Cubitt B, DeWald LE, Radoshitzky SR, Bollinger L, Jahrling PB, Palacios GF, de la Torre JC, Kuhn JH. 2018. Recombinant Lassa virus expressing green fluorescent protein as a tool for high-throughput drug screens and neutralizing antibody assays. Viruses 10:pii: E655. doi: 10.3390/v10110655.

2. Martínez-Sobrido L, Cheng BY, de la Torre JC. 2016. Reverse genetics approaches to control arenavirus. Methods Mol Biol 1403:313-51.

3. Cashman KA, Wilkinson ER, Wollen SE, Shamblin JD, Zelko JM, Bearss JJ, Zeng X, Broderick KE, Schmaljohn CS. 2017. DNA vaccines elicit durable protective immunity against individual or simultaneous infections with Lassa and Ebola viruses in guinea pigs. Hum Vaccin Immunother 13:3010-3019.

4. Sánchez AB, de la Torre JC. 2006. Rescue of the prototypic arenavirus LCMV entirely from plasmid. Virology 350:370-80.

5. Nikisins S, Rieger T, Patel P, Muller R, Günther S, Niedrig M. 2015. International external quality assessment study for molecular detection of Lassa virus. PLoS Negl Trop Dis 9:e0003793.
